# Clinical Efficacy of Application-Linked Stretching Ball as Digital Therapeutics in Plantar Fasciitis

**DOI:** 10.3390/jcm13092722

**Published:** 2024-05-06

**Authors:** Seok Chang Ryu, Dong-Oh Lee, Yoojin Park, Yujeong Shin, Dong Yeon Lee, Min Gyu Kyung

**Affiliations:** 1 BioRobotics Laboratory, Division of Mechanical and Biomedical Engineering, Ewha Womans University, Seoul 03760, Republic of Korea; scryu@ewha.ac.kr; 2 Department of Orthopedic Surgery, SNU Seoul Hospital, Seoul 08703, Republic of Korea; 3 Graduate Program in Smart Factory, Division of Mechanical and Biomedical Engineering, Ewha Womans University, Seoul 03760, Republic of Korea; milkpearl00@ewhain.net (Y.P.); singu1129@ewhain.net (Y.S.); 4 Department of Orthopedic Surgery, Seoul National University College of Medicine, Seoul 03080, Republic of Korea; leedy@snu.ac.kr; 5 Department of Orthopedic Surgery, Kyung Hee University Hospital at Gangdong, Seoul 05278, Republic of Korea

**Keywords:** digital therapeutics, plantar fasciitis, stretching exercises, stretching ball

## Abstract

**Background/Objectives**: This study aimed to evaluate the efficacy of application-linked stretching ball instruments that record the rolling time and force of patients compared with a traditional simple stretching ball. **Methods**: Fourteen participants with plantar fasciitis were divided into a simple massage ball group (group A, n = 8) and an application-linked massage ball group (group B, n = 6). The application-linked massage ball sends information regarding the massages, such as the frequency and force of the massage on the foot, to the application on the patient’s smartphone. All clinical outcomes were evaluated at the beginning of the study and 1-, 2-, and 3-month follow-up. The primary outcome measure was the Manchester–Oxford Foot Questionnaire (MOXFQ) score. **Results**: At the beginning of the study, the initial MOXFQ score was not significantly different between the two groups (*p* = 0.948). At each time point, the MOXFQ score of the whole population did not improve significantly compared to that of the initial state (*p* = 0.131). Generalized estimating equation modeling demonstrated that there was no significant difference in the improvement of the MOXFQ score between groups A and B during follow-up (*p* = 0.826). In addition, no group-by-time interactions were observed (*p* = 0.457). **Conclusions**: The efficacy of an application-linked massage ball for the treatment of plantar fasciitis was not as definite as that of a traditional simple stretching ball in patients whose symptoms persisted for at least six months. Future studies that include patients with acute plantar fasciitis are required.

## 1. Introduction

Plantar fasciitis is one of the most common musculoskeletal diseases, accounting for more than one million annual outpatient visits [1,2]. Patients often report heel pain when first stepping out of bed in the morning. Additionally, this condition can lead to walking and standing problems such as avoiding long distances, altering their gait, needing to stop and rest, avoiding prolonged standing, and avoiding hard surfaces.

The anatomy and biomechanics of the foot play an essential role in the etiology and progression of plantar fasciitis, making a comprehensive understanding of these elements crucial for effective treatment. The plantar fascia is a thick band of connective tissue that extends from the medial tubercle of the calcaneus to the bases of the proximal phalanges. It consists of three bands: the medial, lateral, and central; the central band is the thickest and most commonly involved in plantar fasciitis [3]. Functionally, it acts as a dynamic shock absorber and a stabilizer, helping to distribute the forces exerted on the foot during walking, running, and standing [4]. Biomechanically, the plantar fascia is subjected to considerable stress during the propulsion phases of gait, where it experiences tension as the toes extend and the arch of the foot deepens to provide leverage for pushing off the ground. This mechanical load is crucial for normal foot function but can also predispose the fascia to microtears and inflammation, especially in the context of overuse, improper footwear, or structural abnormalities, such as flatfeet. Many clinical factors are known to be risk factors, such as aging, injury, repetitive or prolonged stimuli, and overweight [4,5,6]. It is becoming more common as the population ages, and many people participate in sports activities [7].

Plantar fasciitis is typically a self-limiting disease, with over 90% of patients finding relief from symptoms within 3 to 6 months through conservative treatment [8]. Currently, there is no definitive procedure or modality for complete treatment. The conservative method is a mainstay of plantar fasciitis treatment, including rest, taking nonsteroidal anti-inflammatory drugs (NSAIDs), orthosis (heel cups and arch support insoles), shoe modification, and stretching exercises (triceps and plantar fascia) [9]. Oral NSAIDs may offer short-term pain relief by suppressing inflammatory mediators via cyclooxygenase pathway inhibition. However, they are not used solely for treatment but rather in conjunction with other modalities to enhance symptom improvement. Orthoses help reduce pain during gait by decreasing the force of the Achilles tendon. However, there are various types available, making it difficult to find one that fits well, and they can also be quite expensive to purchase. Triceps and plantar fascia stretching exercises have been considered as one of the primary methods of conservative treatment compared to any other approach. One simple stretching method that individuals can perform is sitting and rolling a massage ball underfoot, which stretches the plantar fascia. Another method involves bending the knees and extending the forefoot to tighten the plantar fascia, then manually pressing along the plantar fascia to perform the stretch. Patients with plantar fasciitis commonly experience a tight Achilles tendon [10]. When the triceps are tight, they can hinder ankle dorsiflexion and significantly affect the plantar pressure distribution of the entire foot. According to Engkananuwat et al., simultaneous Achilles tendon stretching with plantar fascia stretching for 4 weeks led to superior clinical outcomes when compared with Achilles stretching alone [11]. This stretching can be performed independently or under the guidance of a physical therapist. Previous studies have demonstrated that the clinical outcomes of an independent home stretching protocol and formal physical therapy did not markedly differ for the treatment of plantar fasciitis [12]. However, some patients may continue to experience symptoms even after these treatments. In such cases, methods such as corticosteroid or platelet-rich plasma injections, or extracorporeal shock wave therapy can be used. If more invasive interventions are necessary, surgical treatments, like partial plantar fasciotomy or gastrocnemius lengthening, may be performed [3]. Although various injection therapies and surgical treatments have been reported for these patients, their clinical efficacy has not yet been established [9,13,14].

Recently, digital therapeutics, including those for diabetes and mental health, have been highlighted in behavioral healthcare [15,16]. Prescriptions of digital therapeutics are software-based treatments delivered on smartphones that address the behavioral dimensions of many diseases and conditions [16,17]. This novel approach leverages the widespread adoption of smartphones and wearable technology, proposing a method that not only addresses the physical aspects of the disease but also engages patients in their treatment through interactive and personalized digital interventions. The shift towards digital therapeutics represents a convergence of healthcare and technology, reflecting the broader trend of personalized medicine and the increasing recognition of the importance of patient engagement and behavioral modification in managing chronic conditions. Many of these are authorized by governmental regulators, such as the Food and Drug Administration [16].

The advancement of digital therapeutics has been undoubtedly influenced by technological progress, but their development was further accelerated due to the COVID-19 pandemic. The shift towards isolation changed people’s lifestyles and expanded the concept of non-face-to-face interactions. Instead of visiting hospitals, using digital health technologies such as smartwatches for self-tracking one’s health status has become increasingly popular. However, literature on the clinical efficacy of digital therapeutics in the treatment of musculoskeletal diseases, including plantar fasciitis, is limited.

In our clinical experience, we have encountered many patients with intractable plantar fasciitis in outpatient settings. This led us to question how we could enhance the effectiveness of conservative treatments and how compliant patients actually are with stretching exercises at home, prompting the start of this study. Many runners enjoy using mobile applications to record laps or online competitions with other colleagues during running. Similarly, mobile phone-linked applications that enable monitoring by both patients and clinicians may enhance patient performance in physiotherapy. Numerous mobile applications have already been developed to improve medication adherence [18].

Thus, we sought to identify the clinical efficacy of application-linked stretching ball instruments that record the rolling time and force of patients by comparing them with a traditional simple stretching ball. We hypothesized that an application-linked stretching ball would be more helpful than a traditional simple stretching ball in the treatment of plantar fasciitis.

## 2. Materials and Methods

This prospective study was approved by Seoul National University Hospital Institutional Review Board (IRB number: H-2105-067-1218; approval date: 14 June 2021). Informed consent was obtained from all participants included in the study. This study was conducted in accordance with the principles of the Declaration of Helsinki.

We utilized ChatGPT 4 as a tool, primarily for translating the language used by the authors into English and for assistance in grammatical corrections. This use was limited to enhancing the clarity and readability of our text without affecting the originality, validity, and intellectual integrity of the content.

### 2.1. Study Subjects

**Figure 1:**
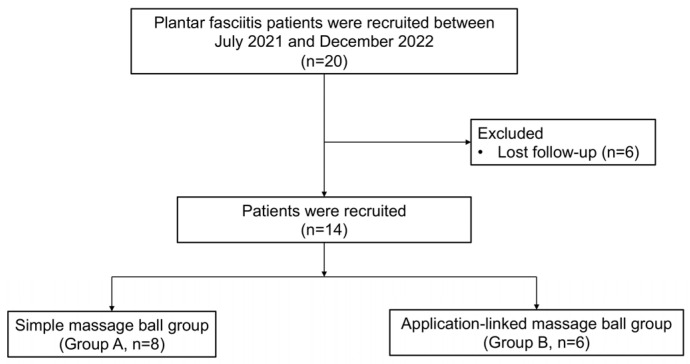
Flowchart illustrating participant selection and grouping.

**Figure 2:**
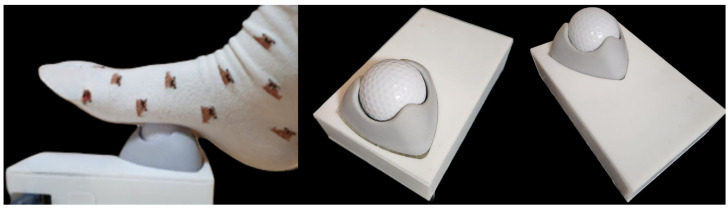
Application-linked massage ball sends information regarding the massage, such as the frequency and the force of massage on the foot, to the application in the patient’s smartphone.

Twenty volunteers with symptoms of plantar fasciitis were recruited from the outpatient clinic of our hospital between July 2021 and December 2022. After screening, the participants were randomly divided into a simple massage ball group (group A) and an application-linked massage ball group (group B) and treated in a single-blind manner (Figure 1). For the assignment to groups A and B, a computer-generated random number table was employed. Six patients were lost to follow-up and were excluded from the final analysis. Therefore, eight patients in group A and six patients in group B were finally included. The application-linked massage ball sends information regarding the massage, such as the frequency and force of massage on the foot, to the application on the patient’s smartphone (Figure 2). We expected that the patients in Group B would be encouraged to be more motivated to undergo ball massaging than the patients in Group A by monitoring their volume of ball massages using an application installed on their smartphones. All study patients were educated using an informative brochure on the general principles of physiotherapy for the treatment of plantar fasciitis, such as rolling or Achilles tendon stretching.

### 2.2. Inclusion Criteria

The inclusion criteria were (1) the presence of plantar heel pain that could be clinically diagnosed as plantar fasciitis by one author; (2) age between 18 and 70 years; (3) pain experienced for >6 months with minimal response to conservative treatment, including oral medication, heel cup, physical therapy, or stretching; and (4) absence of factors, such as stress fracture of the calcaneus, that may mimic plantar fasciitis, as observed in interviews, physical examination, radiographs, and ultrasonography [19].

### 2.3. Exclusion Criteria

The exclusion criteria included patients (1) who declined to participate in the study; (2) with heel pain for any other reason, including a calcaneal stress fracture, infection, tumors, rheumatic enthesopathy, or nerve entrapment; (3) with disorders of the foot or ankle not related to plantar fasciitis; (4) who had received any other injection including steroids in the past 3 months; (5) with systemic diseases or any severe diseases of the heart, liver, or kidney; and (6) with a history of alcohol, drug abuse, or psychiatric history [19].

### 2.4. Stretching Ball Usage

All patients were instructed to use either a simple massage ball or an application-linked massage ball at least three times a day for more than 5 min per session. Additionally, patients in group B were instructed to download applications and run them on their mobile phones. When using the devices, all the patients were instructed to maintain a sitting position comfortably on a chair or sofa and roll stretching balls on their affected soles, including the insertion site of the plantar fascia.

### 2.5. Clinical Outcome Measures

All clinical outcomes were evaluated at the beginning of the study and 1-, 2-, and 3-month follow-up. The primary outcome measure was the Manchester–Oxford Foot Questionnaire (MOXFQ) score [19]. The MOXFQ is a patient-reported outcome measure developed through patient interviews. It has been demonstrated to be a reliable and valid tool for measuring the outcomes of foot surgery and general health [20,21,22]. It contains 16 items comprising three parts: walking/standing problems (seven items), foot pain (five items), and social interactions (four items). Each item is scored from 0 to 4, with 4 meaning most severe.

### 2.6. Statistical Anaylsis

The distribution of data was evaluated using the Kolmogorov–Smirnov test. Fisher’s exact test was used to analyze categorical variables, such as sex ratio. The Mann–Whitney U test was used to compare the initial and last follow-up statuses in the whole population and each group.

To analyze the association between the application-linked stretching ball and MOXFQ scores at each time point (initial state and at one, two, and three months), a generalized estimating equation (GEE) method was used. A *p*-value < 0.05 was considered significant. All statistical analyses were performed using SPSS version 22 (IBM Corp., Armonk, NY, USA).

Initially, the sample size was set at 20 participants per group based on previous research [19]. The effect size was calculated as 10, and it was determined that 18 patients per group were needed to achieve a power of 0.8, with an alpha error of 0.05. Considering the dropout rate of approximately 20%, the study was designed to include 40 patients (20 patients in Group A and B, respectively). However, this study was conducted during the peak of the COVID-19 pandemic, which made recruiting participants very difficult. As a result, we were only able to recruit half the intended number, i.e., 20 participants, during the study period. Of these, 6 were lost to follow-up, resulting in the experiment being conducted with 8 participants in group A and 6 in group B.

## 3. Results

Demographic data of the participants are demonstrated in Table 1 and summarized in Table 2. There were no statistically significant differences in age, sex ratio, or body weight between the two groups.

The recorded average duration of usage per session for the application-linked massage ball among participants in Group B was 24.8 ± 20.2 min, and the average daily frequency of use was 1.9 ± 0.9 times.

**Figure 3:**
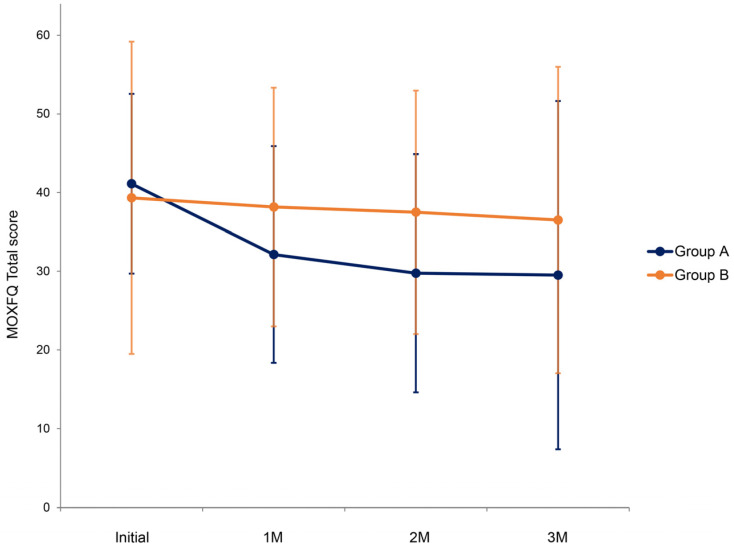
Comparison of total Manchester–Oxford Foot Questionnaire (MOXFQ) score between groups during follow-up. Group A is a simple massage ball group and Group B is an application-linked massage ball group.

At the beginning of the study, the initial total MOXFQ scores showed no significant difference between the two groups (*p* = 0.948) (Table 3 and Figure 3). At each time point, the total MOXFQ score of the whole population did not improve significantly compared to that of the initial state (*p* = 0.131). GEE modeling demonstrated no significant difference in the improvement of the total MOXFQ score between groups A and B during follow-up (*p* = 0.826). No group-by-time interactions were observed (*p* = 0.457).

**Figure 4:**
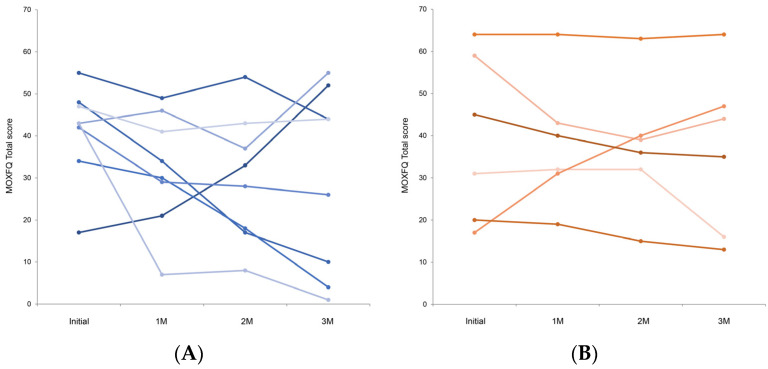
(**A**) Individual total Manchester–Oxford Foot Questionnaire (MOXFQ) scores for participants in Group A during follow-up. (**B**) Individual total Manchester–Oxford Foot Questionnaire (MOXFQ) scores for participants in Group B during follow-up.

The MOXFQ total scores for individual participants in both groups generally decreased over the follow-up period compared with the initial state (Figure 4A,B). However, one participant in each group exhibited a consistent increase in their scores.

**Figure 5:**
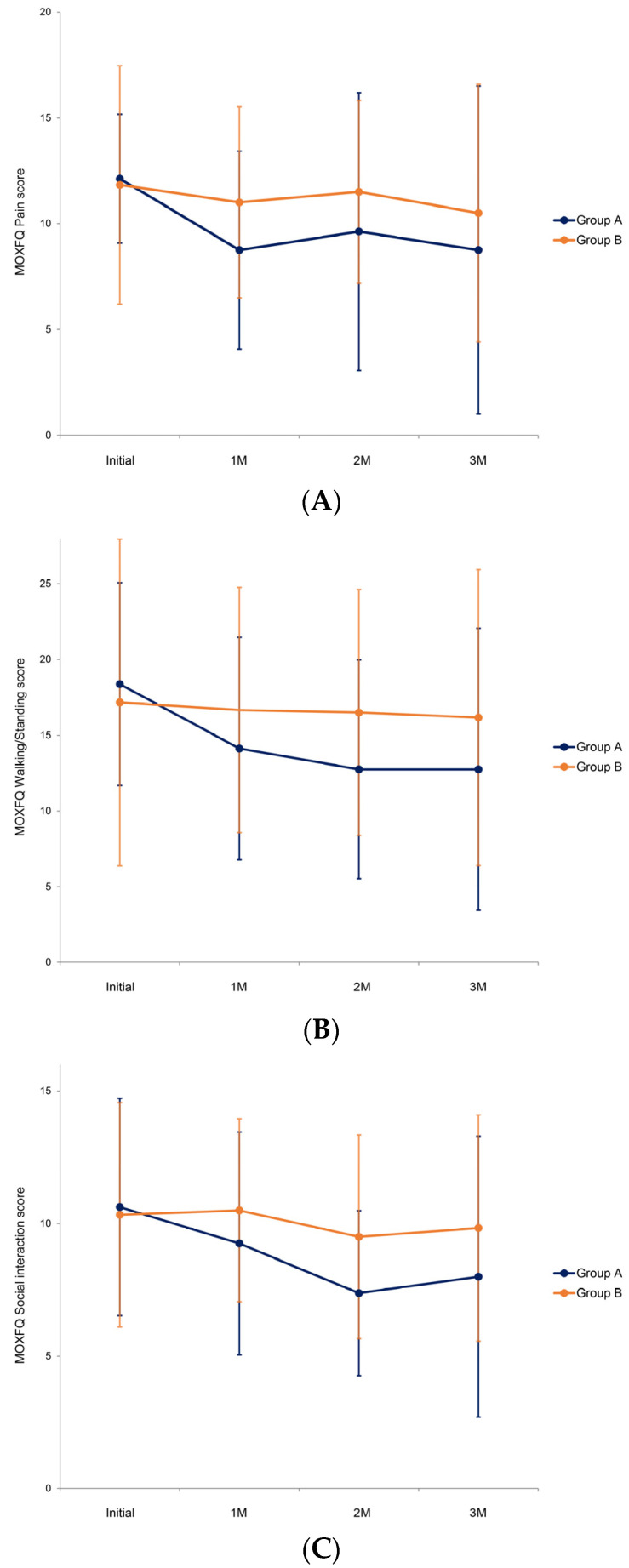
(**A**) Comparison of Manchester–Oxford Foot Questionnaire (MOXFQ) pain scores between groups during follow-up. (**B**) Comparison of Manchester–Oxford Foot Questionnaire (MOXFQ) walking/standing scores between groups during follow-up. (**C**) Comparison of Manchester–Oxford Foot Questionnaire (MOXFQ) social interaction scores between groups during follow-up. Group A is a simple massage ball group and Group B is an application-linked massage ball group.

Breaking down the total MOXFQ score into pain, walking/standing, and social interaction scores, we observed that, at the beginning of the study, the initial MOXFQ pain scores showed no significant difference between the two groups (*p* = 0.650) (Figure 5A). Throughout the study, the pain scores of the whole population did not improve significantly compared with the initial state (*p* = 0.260). GEE modeling revealed no significant difference in the improvement of the MOXFQ pain scores between groups A and B during follow-up (*p* = 0.990).

The initial MOXFQ walking/standing scores showed no significant difference between the two groups (*p* = 0.897) (Figure 5B). Throughout the study, the walking/standing scores of the whole population did not improve significantly compared with the initial state (*p* = 0.139). GEE modeling revealed no significant difference in the improvement of the MOXFQ walking/standing scores between groups A and B during follow-up (*p* = 0.750).

The initial MOXFQ social interaction scores showed no significant difference between the two groups (*p* = 0.845) (Figure 5C). During the follow-up, the social interaction scores of the whole population did not improve significantly compared with the initial state (*p* = 0.110). GEE modeling revealed no significant difference in the improvement of the MOXFQ social interaction scores between groups A and B during follow-up (*p* = 0.819).

No complications related to the stretching ball instruments or simple stretching balls were reported throughout the study period.

## 4. Discussion

Our study showed that patients with plantar fasciitis who used application-linked stretching ball instruments did not show more favorable clinical outcomes than those who used simple traditional ball instruments during short-term follow-up.

Plantar fasciitis is characterized by pain in the plantar heel that is exacerbated by weight-bearing activities [23]. This is related to tendinopathy, which is considered a degenerative pathology, rather than a primary inflammatory condition [23]. Many cases resolve within one year, if patients follow the general principles of conservative treatment, such as rest, stretching, and insoles [23].

Some patients sustain symptoms for more than one year and experience intractable plantar fasciitis. Numerous risk factors, such as aging, obesity, repetitive injury, or prolonged stimuli, may attribute to these chronic symptoms [4,5,6]. Relieving symptoms quickly is important because plantar fasciitis reduces quality of life. Previous studies have demonstrated that patients with plantar heel pain have poorer generic and foot-specific health-related quality of life [24]. It has also been reported that plantar heel pain leads to affected individuals not only having poorer foot and broader physical function, but also poorer social functioning [24]. In these cases, clinicians try other modalities, such as injection therapy or surgical treatment, which have been reported to date [13,25]. These include many other treatment methods reported to cure plantar fasciitis, such as Botox, steroids, and extracorporeal shockwave therapy [25,26].

However, the clinical efficacy of these modalities has not yet been determined, although several studies have reported good clinical outcomes with each modality. We suspect that the compliance of patients with prolonged plantar fasciitis might be somewhat different from that of patients who show earlier recovery from plantar fasciitis. Barnes et al. reported that clinical and functional characteristics of people with chronic and recent onset plantar heel pain are different in that chronic plantar heel pain is associated with selective weakness of foot and ankle muscle groups [27]. In fact, stretching exercises are not encouraged and are not monitored by a physical therapist or clinician because rehabilitation exercises are mainly performed in the homes of the patients during the follow-up period in many cases.

Therefore, we postulated that a considerable number of cases, which could have spontaneously resolved, may progress to chronic intractable plantar fasciitis because patients did not follow the general rules of stretching exercises, especially from the perspective of exercise frequency and strength. Anecdotally, we found that patients who received a paper sheet on which they were supposed to record the amount of stretching exercises showed favorable clinical outcomes in our pilot study. However, paper sheets have limitations in terms of inconvenience, delivery, recording, and the possibility of loss. Moreover, we could not expect patients with low compliance with stretching exercises to readily record their performance on paper sheets. Therefore, we decided to conduct this study using an application-linked ball instrument.

Previous studies have shown that a web-based exercise programming system can improve home exercise adherence and confidence in people with musculoskeletal conditions [28]. However, the literature on using such web or application-based interventions to enhance patient adherence during the conservative treatment of musculoskeletal diseases is limited. In contrast, many applications have been developed for medical conditions, such as cardiovascular disorders, where regular follow-ups are challenging due to distance, ensuring proper medication adherence and reminding patients to continue their medications [18]. The efficacy of these applications has been well documented [29]. Recently, the rapid advancement of wearable devices, like smartwatches, has further accelerated such research. However, the difference between conservative treatment for musculoskeletal conditions and medication adherence for medical diseases is significant because the effects of prescribed stretching exercises do not appear quickly. Therefore, we believe that the patients’ loyalty to the doctor’s prescription of stretching exercises, along with consistent adherence to performing these exercises, is crucial for successful treatment.

Patient compliance seems to indirectly manifest in the results of our study as well. Although the efficacy of the application-linked massage ball was not distinctly evident, the final follow-up scores using stretching techniques in both groups are still higher when compared with the MOXFQ scores of other modalities, such as corticosteroid or polydeoxyribonucleotide injections for plantar fasciitis [19]. Both groups were instructed to perform stretching exercises at least three times a day for more than 15 min per session. However, while it is unclear how much Group A complied, Group B, despite knowing their sessions were being recorded, averaged about 1.9 sessions per day lasting approximately 24.8 min each.

Since both groups showed no significant clinical improvement at the final 3 months follow-up, we could not determine whether self-monitoring devices, such as ball machines, had additional effects in improving the clinical outcomes of patients with plantar fasciitis. One possible reason is that 30% of the recruited participants were lost to follow-up, obscuring any significant differences between the groups. Additionally, the study was conducted during the COVID-19 period, which made initial enrollment challenging and is likely to have contributed to the high rate of follow-up loss. Due to these reasons, it was difficult to analyze the clinical outcome differences based on sex and age group stratification within the group. There have been several reports on the differences in plantar fascia demographics. According to Huerta and García, males had a 0.42 mm increased thickness in the plantar fascia 1 cm proximal to the insertion compared with females in asymptomatic subjects [30]. Meanwhile, Granado et al. reported that, while healthy individuals show similar plantar fascia thickness [31], those with plantar fasciitis develop thicker fascia in males [31,32]. In addition to these anatomical differences, females with plantar fasciitis have demonstrated worse health-related quality of life regarding foot pain, foot function, footwear, and general foot health compared to males [33]. Although there was no significant difference in the proportion of females between Group A and B, the relatively higher proportion of females in Group B may have contributed to the less meaningful effect of the application-linked massage ball.

Zhang et al. investigated anatomical features of plantar fasciitis in various age groups and reported that the thickness of sagittal plantar fascia and the high signal intensity of plantar fascia and surrounding soft tissue were different between age groups [34]. The other study revealed higher prevalence of disabling plantar heel pain in older age groups when compared with middle-aged groups [7]. Therefore, incorporating a greater number of patients in each group and conducting detailed subgroup analysis could enhance our understanding of the variables impacting clinical outcomes. Nevertheless, based on our results, we can assume that patients referred to tertiary medical centers, such as our institution, who have sustained their symptoms for at least 6 months, may need further modalities, such as injection or surgical treatment.

Previous studies have reported that individuals with chronic plantar fasciitis demonstrate significantly greater vascularity and thickened fascia [35]. Given the reports of improved pain scores following selective embolization of abnormal vascular vessels found in such chronic intractable states, it suggests that the characteristics for acute and chronic plantar fasciitis may differ [36,37]. From this perspective, the application-linked massage balls might have an additional effect in patients whose symptoms lasted for less than six months, such as the time until recovery of symptoms in the treatment of plantar fasciitis, compared with the traditional massage ball. Therefore, in future studies, it would be beneficial to divide patients into two groups: those in the acute phase within six months and those with chronic plantar fasciitis. Comparing the efficacy of an application-linked massage ball between these groups could provide valuable insights.

Remote-network-based fitness programs became popular after the COVID pandemic. Such programs can monitor individual competence and provide convenient feedback without contact, similar to digital therapeutic applications. Digital therapeutics can fill the gap between unmonitored home-based curative exercises and clinicians’ observations. From this perspective, we believe that application-linked stretching ball instruments could help in earlier recovery from plantar fasciitis for certain patients.

Although our study could not demonstrate a positive effect of the application-linked massage ball for treating plantar fasciitis, it does highlight current limitations and can provide valuable ideas for future research. Specifically, while we were able to collect data by recording the rolling time and force exerted by patients using the device, we failed to provide them with direct positive feedback and encouragement based on this data. Whereas older wearable devices were akin to pedometers, simply tracking daily steps, modern smart wearable devices can prompt users with notifications to walk more towards a daily goal, or even allow users to share data with peers using the same application to foster competition and help achieve goals. Therefore, a future development of a smart massage ball could not only record data but also provide direct feedback through integration with a smartwatch, potentially enhancing its positive effects.

Our study has some limitations. First, the study was performed with a small sample size and in a single-blinded manner. Second, patients who used iPhones could not use the application-linked machine because the applications used in this study were made only for Android phone, owing to a shortage of funds. However, we believe that this could reduce the possibility of selection bias that might occur when we include both users of Android phones and iPhones, because their compliance may not be similar. Third, we could not control the rehabilitation protocol completely because the general physiotherapy protocols for the treatment of plantar fasciitis vary. Nonetheless, we provided both groups with an informative brochure containing text and simple illustrations on the general principles of physiotherapy for the treatment of plantar fasciitis, such as rolling or Achilles tendon stretching. Previous studies have suggested that distributing home therapy exercises in a video-format is superior to paper handouts for at-home hand and wrist rehabilitation [38]. It was noted that patients in the video group utilized more exercises and had higher confidence in completing them correctly after starting the exercise program. Therefore, in subsequent studies, educating patients via a video format on rolling or Achilles tendon stretching might yield better results.

## 5. Conclusions

The efficacy of an application-linked massage ball for the treatment of plantar fasciitis was not as definite as that of a traditional simple stretching ball in patients whose symptoms persisted for at least 6 months. Future studies that include patients with acute plantar fasciitis are required.

## Figures and Tables

**Table 1:** Participants’ individual demographic data.

Patient No.	Group	Sex	Age (Year)	Body Weight (kg)
1	A	M	54	86
2	A	F	28	65
3	A	F	66	70
4	A	M	70	68
5	A	F	57	62
6	A	F	30	53
7	A	F	62	60
8	A	M	28	87
9	B	F	53	56
10	B	F	63	62
11	B	F	66	57
12	B	F	60	62
13	B	F	66	63
14	B	M	61	95

Note: The patient numbers presented in the table above do not represent the actual order in which groups were assigned during the study; rather, they were rearranged for convenience to clearly delineate Group A and Group B.

**Table 2:** Summary of the participants’ demographic data.

	Group A (n = 8)	Group B (n = 6)	*p* Value
Age, year	49.4 (28.0–70.0)	61.5 (53.0–66.0)	0.345
Sex, number	Men 3, Women 5	Men 1, Women 5	0.580
Body weight, kg	68.9 (53.0–87.0)	65.8 (56.0–95.0)	0.491

Data are presented as mean (range).

**Table 3:** Manchester–Oxford Foot Questionnaire (MOXFQ) scores between groups during follow-up.

	Initial	1M	2M	3M
Group A (n = 8)				
Total, points	41.1 ± 11.4	32.1 ± 13.8	29.8 ± 15.1	29.5 ± 22.1
Pain, points	12.1 ± 3.0	8.8 ± 4.7	9.6 ± 6.6	8.8 ± 7.8
Walking/Standing, points	18.4 ± 6.7	14.1 ± 7.3	12.8 ± 7.2	12.8 ± 9.3
Social interaction, points	10.6 ± 4.1	9.3 ± 4.2	7.4 ± 3.1	8.0 ± 5.3
Group B (n = 6)				
Total, points	39.3 ± 19.8	38.2 ± 15.2	37.5 ± 15.5	36.5 ± 19.5
Pain, points	11.8 ± 5.6	11.0 ± 4.5	11.5 ± 4.3	10.5 ± 6.1
Walking/Standing, points	17.2 ± 10.8	16.7 ± 8.1	16.5 ± 8.1	16.2 ± 9.8
Social interaction, points	10.3 ± 4.2	10.5 ± 3.4	9.5 ± 3.8	9.8 ± 4.3

Data are presented as mean ± standard deviation.

## Data Availability

The datasets generated during and/or analysed during the current study are available from the corresponding author on reasonable request.
